# The Urease Inhibitor NBPT Negatively Affects DUR3-mediated Uptake and Assimilation of Urea in Maize Roots

**DOI:** 10.3389/fpls.2015.01007

**Published:** 2015-11-19

**Authors:** Laura Zanin, Nicola Tomasi, Anita Zamboni, Zeno Varanini, Roberto Pinton

**Affiliations:** ^1^Department of Agricultural and Environmental Sciences, University of UdineUdine, Italy; ^2^Department of Biotechnology, University of VeronaVerona, Italy

**Keywords:** DUR3 transporter, urea acquisition, high affinity transport, *N*-(*n*-butyl) thiophosphoric triamide, nitrogen nutrition, *Zea mays*, ammonium transporter, urea metabolism

## Abstract

Despite the widespread use of urease inhibitors in agriculture, little information is available on their effect on nitrogen (N) uptake and assimilation. Aim of this work was to study, at physiological and transcriptional level, the effects of *N*-(*n*-butyl) thiophosphoric triamide (NBPT) on urea nutrition in hydroponically grown maize plants. Presence of NBPT in the nutrient solution limited the capacity of plants to utilize urea as a N-source; this was shown by a decrease in urea uptake rate and ^15^N accumulation. Noteworthy, these negative effects were evident only when plants were fed with urea, as NBPT did not alter ^15^N accumulation in nitrate-fed plants. NBPT also impaired the growth of *Arabidopsis* plants when urea was used as N-source, while having no effect on plants grown with nitrate or ammonium. This response was related, at least in part, to a direct effect of NBPT on the high affinity urea transport system. Impact of NBPT on urea uptake was further evaluated using lines of *Arabidopsis* overexpressing *ZmDUR3* and *dur3*-knockout; results suggest that not only transport but also urea assimilation could be compromised by the inhibitor. This hypothesis was reinforced by an over-accumulation of urea and a decrease in ammonium concentration in NBPT-treated plants. Furthermore, transcriptional analyses showed that in maize roots NBPT treatment severely impaired the expression of genes involved in the cytosolic pathway of ureic-N assimilation and ammonium transport. NBPT also limited the expression of a gene coding for a transcription factor highly induced by urea and possibly playing a crucial role in the regulation of its acquisition. This work provides evidence that NBPT can heavily interfere with urea nutrition in maize plants, limiting influx as well as the following assimilation pathway.

## Introduction

Urea is the most frequently used nitrogen (N) fertilizers in the world with annual amount of over 50 million tons accounting for more than 50% of the world N fertilizer consumption (International Fertilizer Industry Association, 2008). The incredible increase in urea fertilizer use during the last decades is mainly due to its competitive price and the high N content (46% of mass), that allow reducing transport and distribution costs (Miller and Cramer, 2004).

Although experimental evidence reported the ability of plants to use urea *per se* when supplied through leaf application (Wittwer et al., 1963; Nicolaud and Bloom, 1998; Witte et al., 2002), a common agronomic practice is to supply urea to the crops by soil fertilization. Besides using inorganic N sources, plants, including crops, have been shown to be able to take up intact urea (for review, see Kraiser et al., 2011; Nacry et al., 2013). In particular, maize plants possess dedicated transmembrane transport systems in root cells for the acquisition of urea with high and low affinity, mediated by a DUR3 transporter and aquaporins, respectively (Gaspar et al., 2003; Gu et al., 2012; Zanin et al., 2014; Liu et al., 2015; Yang et al., 2015).

In the soil solution the stability of urea is strictly dependent on the activity of the microbial urease, a nickel-dependent enzyme ubiquitously expressed in microorganisms and released into soil (Watson et al., 1994). Moreover urease activity can persist in the soil even after the decay of the microorganisms (Watson et al., 1994). This enzyme catalyzes the hydrolysis of urea into ammonium and carbon dioxide and its activity is proportional to the microbial biomass, which in turn depends on the organic matter amount and the water content of the soil. Ammonium could remain in this form as exchangeable cation or volatilized in form of ammonia; it could also serve as a substrate for nitrification process being transformed into nitrate. Thus, at least for short periods of time, urea fertilization may result in a simultaneous exposure of plant roots to urea, ammonium and nitrate (Mérigout et al., 2008b).

Mainly due to ammonia volatilization and nitrate leaching, the rapid hydrolysis of urea would lead to a decreased N availability for plant nutrition and to a lower use efficiency of urea fertilizers (Zaman et al., 2008). So one of the most used strategies to reduce ammonia emissions from urea fertilizer is to apply urease inhibitors. Besides slowing urea hydrolysis, these molecules allow the diffusion of urea far away from the application site favoring its uptake as an intact molecule by the plant roots.

The most promising and tested soil urease inhibitor is the NBPT(trade name Agrotain^®^), whose activity is associated with the conversion to its oxidized form (Watson, 2005). NBPT is a structural analog of urea (Medina and Radel, 1988) acting with mixed inhibition on urease activity (increased *K*m and decreased *V*max; Juan et al., 2009). Molecular dynamic calculations showed that NBPT coordinates both nickel atoms of the urease active site and binds the oxygen atom of the urea-derived carbamate (Manunza et al., 1999).

It is not unusual to find marketing formulations containing urea in combination with urease inhibitor (Watson, 2005). Experimental evidence has been provided showing that the activity of urease inhibitors could be affected by environmental factors such as pH (Hendrickson and Douglass, 1993), temperature (Hendrickson and O’Connor, 1987), and soil moisture content (Sigunga et al., 2002; Clough et al., 2004).

Limited information is available on the physiological effects of NBPT in plants (Watson and Miller, 1996; Cruchaga et al., 2011). It has been reported that some species showed visible symptoms of toxicity when plants were treated with urea and NBPT with the transient development of leaf scorches and necrotic leaf margins (Watson and Miller, 1996; Artola et al., 2011; Cruchaga et al., 2011). Cruchaga et al. (2011) reported that NBPT is taken up by pea and spinach roots and translocated to the leaves; thus NBPT can conceivably inhibit the activity of endogenous leaf and root urease (Watson and Miller, 1996; Artola et al., 2011; Cruchaga et al., 2011; Ariz et al., 2012). Moreover glutamine synthetase activity and amino acid level are reduced in presence of NBPT (Artola et al., 2011; Cruchaga et al., 2011). Altogether these results showed that the urease inhibitor compromised the use of urea as a source of N for plants, but there is still a lack of knowledge on the physiological and molecular aspects of NBPT effects on the acquisition of this N source.

The aim of the current research was to study the short-term effects of NBPT on the capacity of maize plants to acquire urea. Previous studies from our group described *in vivo* the high affinity transport system of urea in maize roots and showed that urea quickly induce its acquisition (Zanin et al., 2014). Therefore, in the present work the action of NBPT was studied on the functionality of the inducible component of the high affinity influx system. Physiological data were supported by analysis of changes in the transcription of genes known to be modulated by urea.

## Materials and Methods

### Plant Material and Growth Conditions of Maize

Maize seeds (*Zea mays* L., inbred line PR33T56, Pioneer Hybrid Italia S.p.A.) were germinated over aerated 0.5 mM CaSO_4_ solution in a dark growth chamber at 25°C. After 3 days, the seedlings were transferred into an aerated hydroponic system in a controlled climatic conditions: day/night photoperiod, 16/8 h; light intensity, 220 μmol m^-2^s^-1^; temperature (day/night) 25/20°C; relative humidity, 70–80%. After 2 days (5-day-old) plants were transferred to a nutrient solution containing (μM): KCl 5; CaSO_4_ 500; MgSO_4_ 100; KH_2_PO_4_ 175; NaFe-EDTA 20; H_3_BO_3_ 2.5; MnSO_4_ 0.2; ZnSO_4_ 0.2; CuSO_4_ 0.05; Na_2_MoO_4_ 0.05. Nitrogen was added in form of: 0.5 mM CO(NH_2_)_2_ (*Urea* treatment); 0.5 mM Ca(NO_3_)_2_ (*Nitrate* treatment); 0.5 mM (NH_4_)_2_SO_4_ (*Ammonium* treatment). As control, plants were exposed to a N-free nutrient solution (*Control* treatment). For experiments reported in Supplementary Table S1, two additional treatments were used: 0.5 mM CO(NH_2_)_2_ + 0.5 mM Ca(NO_3_)_2_ (*Urea* + *Nitrate* treatment) and 0.5 mM CO(NH_2_)_2_ + 0.5 mM (NH_4_)_2_SO_4_ (*Urea* + *Ammonium* treatment).

For ^15^N experiments, maize plants were grown in hydroponic conditions as described above and treated with ^15^N-labeled sources supplied to N-free nutrient solution in form of: 0.5 mM CO(^15^NH_2_)_2_ (98 atom% ^15^N, in ^15^N-urea containing treatments), 0.5 mM Ca(^15^NO_3_)_2_ (98 atom% ^15^N, in ^15^N-nitrate containing treatments) or 0.5 mM (^15^NH_4_)_2_SO_4_ (98 atom%, in ^15^N-ammonium containing treatment; ISOTEC^®^ Stable Isotopes, Sigma–Aldrich, Milano, Italy).

The urease inhibitor NBPT (Apollo Scientific Ltd, UK) was applied to nutrient solution at 0.5% of the weight of urea, which is the concentration used in the commercial formulation of NBPT-urea fertilizer. Preliminary experiments showed that an effect on urea uptake could be observed also halving the NBPT concentration, however, this concentration would not guarantee a proper control of urease activity (and preservation of urea) for an adequate time span (Watson and Miller, 1996). Thus, in our experiments 0.897 μM NBPT were present in the nutrient solution of: *Urea* + *NBPT* treatment, *Nitrate* + *NBPT* treatment, and *Control* + *NBPT* treatment. The pH of solution was adjusted to pH 6.0 with potassium hydroxide (KOH). Nitrogen sources and/or NBPT were supplied to nutrient solution after 1 h from the beginning of the light phase (T_0_ = 0 h of treatment).

A morphological evaluation was performed on 5-day-old maize plants exposed for 7 days to the different N treatments; while physiological and transcriptional analyses were performed on 5-day-old maize plants exposed up to 24 h to the different N treatments. After 0, 2, 4, 8, 12, and 24 h of treatment, pool of six plants for each sample were analyzed immediately for physiological experiments or stored at –80°C until further processing for molecular works.

### Morphological Evaluation of Maize Roots

**FIGURE 1:**
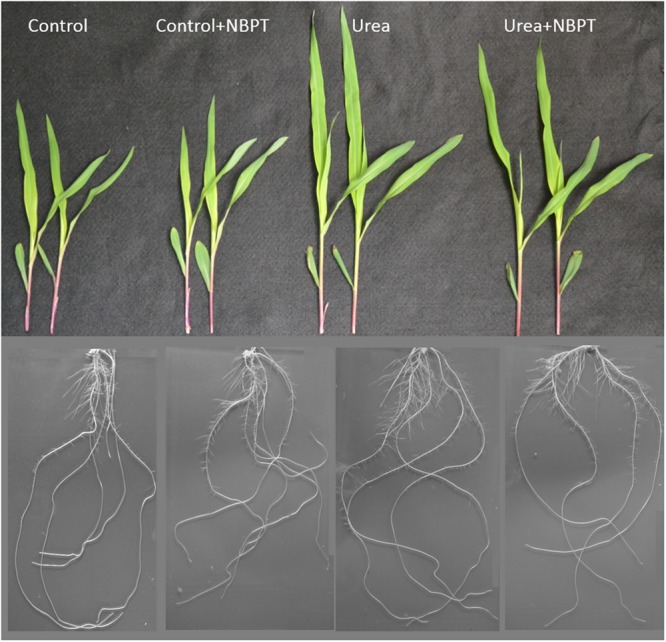
**Morphological effect of urea and NBPT on the shoot and root systems of maize plants.** Five-day-old maize plants were grown hydroponically for 7 days in a nutrient solution supplied with 0.5 mM urea in presence or absence of 0.897 μM NBPT (*Urea* + *NBPT* treatment or *Urea* treatment, respectively) or exposed to a N-free nutrient solution, with or without NBPT (*Control* + *NBPT* treatment or *Control* treatment, respectively). For each treatment, a picture of two representative shoots and a picture of a root system are shown.

To evaluate the biomass production and the root morphology, long term experiments were carried out feeding 5-day-old maize seedlings with different N-sources for 7 days; to ensure constant N-availability, nutrient solutions were renewed daily. At the end of the experiment, shoots and roots of plants were collected and weighted; photos of the root systems were taken (representative samples are shown in **Figure 1**).

The analyses of root systems were performed using “GiA Roots” software (Galkovskyi et al., 2012) based on three independent biological replicates. According to Galkovskyi et al. (2012), descriptions of traits are reported in the legend of Supplementary Table S1.

### ^15^N-accumulation in Maize Tissues

For ^15^N experiments, approximately 1 mg of dried root and leaf tissues was transferred into a tin capsule for measurement of δ^15^N, as described by Zamboni et al. (2014). The ^15^N content of each sample was determined through isotope ratio mass spectrometry analysis coupled with an elemental analyzer (Delta V IRMS, Thermos Scientific, Waltham, MA, USA).

### Measurement of Net High-affinity Urea Uptake in Intact Roots of Maize Plants

Roots of intact seedlings were immersed for 10 min, a time span during which uptake maintained a linear trend, in 40 ml of a constantly stirred and aerated solution containing 500 μM CaSO_4_ and 200 μM urea. To evaluate the direct effect of NBPT on net-uptake rate of urea, 0.897 μM NBPT were added to solution containing 500 μM CaSO_4_ and 200 μM urea. Net uptake rates were measured following the protocol described in Zanin et al. (2015b) and expressed as urea depletion from the solution per unit of time (μmol urea g^-1^ root FW h^-1^).

### Measurement of Urea and Ammonium Content in Maize Tissues

For urea and ammonium determination, leaves and roots of maize were sampled and processed as described by Witte et al. (2002). The urea content was quantified using the diacetyl monoxime and thiosemicarbazide reagents and measuring the absorbance at 527 nm. The ammonium quantification was performed using the Barthelot reagent (EN ISO 11732) on a Skalar San^++^ Autoanalyzer (Breda, Netherlands), the absorbance was determined at 660 nm.

### Real Time RT-PCR Analyses

RNA extractions were performed using the Invisorb Spin Plant RNA kit (Stratec Molecular, Berlin, Germany) as reported in the manufacturer’s instructions. Maize roots (70 mg) were homogenized in liquid N and the powder was mixed with 900 μl of DCT solution and dithiothreitol according to the supplier’s instructions. The RNA was evaluated in an agarose/formaldehyde gel and quantified by spectrophotometer Nanodrop 2000 instrument (Thermo Scientific, Wilmington, DE, USA).

Total RNA was treated with 1 U μg^-1^ RNA of Deoxyribonuclease I (Sigma–Aldrich, Milano, Italy) and cDNA was synthesized from 1 μg of RNA following the application protocol of the manufacturers [42°C for 1 h with 1 pmol of Oligo d(T)_23_VN, Sigma–Aldrich, Milano, Italy; 15 U Prime RNase Inhibitor, Eppendorf, Hamburg, Germany; 10 U M-MulV RNase H^-^, Finnzymes, Helsinki, Finland]. After RNA digestion with 1 U RNase A (USB, Cleveland, OH, USA) for 1 h at 37°C, gene expression analyses were performed by adding 0.16 μl of the cDNA to the real-time RT-PCR complete mix, FluoCycle^TM^ sybr green (20 μl final volume; Euroclone, Pero, Italy), in a DNA Engine Opticon Real Time PCR Detection (Biorad, Hercules, CA, USA).

At the beginning of the experiment (0 h of treatment) and after 2, 8, and 24 h of treatment, the transcript amounts of *ZmDUR3* (coding for a high affinity urea transporter), *ZmUrease* (for urease enzyme), *ZmZFP16-1* (for a zinc finger protein), *ZmGln1-5* (for a glutamine synthetase), *ZmAsnS4* (for AsnS4), *ZmAMT1;3* (for an AMT) were analyzed. The primers were designed using Primer3 software (Koressaar and Remm, 2007; Untergrasser et al., 2012) and they were synthesized by Sigma–Aldrich (Milano, Italy; Supplementary Table S2). The analyses of real-time result were performed using Opticon Monitor 2 software (Biorad) and R (version 2.9.0^1^) with the qPCR package (version 1.1-8^2^). Efficiencies of amplification were calculated following the authors’ indications (Ritz and Spiess, 2008). Real-time RT-PCR results were validate using two housekeeping genes, *ZmTUA and ZmGAPDH*; in the present work the expression patterns relative to *ZmGAPDH* are shown. Data were normalized with respect to the transcript level of the housekeeping genes using the 2^-ΔΔ^*^C^*^T^ method, where ΔΔ*C*_T_ = (*C*_T,Target_ – *C*_T,HK_)_Time x_ – (*C*_T,Target_ – *C*_T,HK_)_Time 0_ (Livak and Schmittgen, 2001).

### Plant Material and Growth Conditions of *Arabidopsis*

*Arabidopsis thaliana* plants [wild type Col-0, *atdur3-3* line and two *ZmDUR3*-overexpressing lines (Col-0 + *35sCaMV:ZmDUR3*- and *atdur3-3* + *35sCaMV:ZmDUR3*-overexpressing lines), Zanin et al., 2014] were grown on axenic conditions. Surface-sterilized seeds were grown on agar plates as described by Kojima et al. (2007). Plants were grown on modified half-strength Murashige and Skoog (MS) medium without N, supplemented with 1 μM NiCl_2_ and 50 μM KNO_3_. Either 0.5 mM NH_4_NO_3_ or 0.5 mM urea or 3.0 mM urea were added as N sources. *Arabidopsis* plants were cultured for 18 days in a growth chamber under controlled climatic conditions: day/night photoperiod, 8/16 h; light intensity, 220 μmol m^-2^s^-1^; temperature (day/night) 22/20°C; relative humidity, 70–80%.

### Statistical Analyses

Physiological and transcriptional analyses were performed on three independent experiments (*n* = 3); for each sample a pool of six plants was used.

Statistical significance was determined by one-way analysis of variances (ANOVA) using Student–Newman–Keuls test (*n* = 3, *P* < 0.05). Statistical analyses were performed using SigmaPlot 12.0 software.

## Results

### Morphological Traits of Maize Plants Treated with Urea and NBPT

**FIGURE 2:**
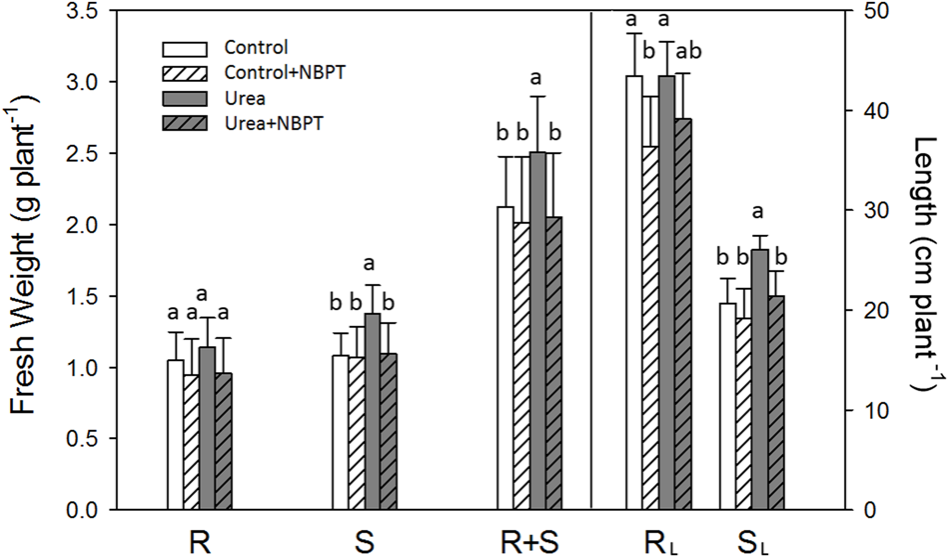
**Effect of urea and NBPT on biomass and length of maize roots and shoots.** Growth conditions as in **Figure 1**. FWs were based on the whole root system (R, root), whole shoot part (S, shoot) or whole plant (R + S, root and shoot). Length of primary root (R_L_, root length) and of main leaf (S_L_, shoot length) were measured. Data are means of three independent biological replicates +SD (Student–Newman–Keuls method ANOVA, *n* = 3, *P* < 0.05).

Maize plants grown for 1 week under hydroponic conditions in presence of urea (*Urea* treatment) showed an increased shoot biomass and length in comparison to *Control* plants grown without N supply (**Figures 1** and **2**). Using “GiA roots” software, a quantification of root system parameters confirmed that urea promoted whole root development, with a significant increase in the *Maximum Number of Roots, Network area, Network perimeter, Network surface area*, and *Network length* (Supplementary Table S1).

For comparison, plants fed with other N sources, such as ammonium (*Ammonium* treatment), nitrate (*Nitrate* treatment), urea and ammonium (*Urea* + *Ammonium* treatment) or urea and nitrate (*Urea* + *Nitrate* treatment) were analyzed. The ammonium supply (*Ammonium* and *Urea* + *Ammonium* treatments) strongly limited the root growth negatively impacting most morphometric parameters. On the other hand, in presence of nitrate (*Nitrate* treatment) the development of root system was just slightly higher than in *Control* plants; when nitrate was applied in conjunction with urea (*Urea* + *Nitrate* treatment) the highest morphometric values were recorded (Supplementary Table S1).

The stimulatory action of urea on plant development was severely limited by addition of the urease inhibitor NBPT to the nutrient solution. In comparison to *Urea* treated plants, *Urea* + *NBPT* treated plants showed a reduction in shoot weight, shoot length (**Figures 1** and **2**), *Network area, Network perimeter*, and *Network surface area* (Supplementary Table S1).

### Effect of NBPT on ^15^N Accumulation in Maize Plants

The effect of NBPT on the acquisition of urea and nitrate was evaluated measuring ^15^N accumulation in roots and shoots of plants after 24 h of treatment with different ^15^N-sources.

**FIGURE 3:**
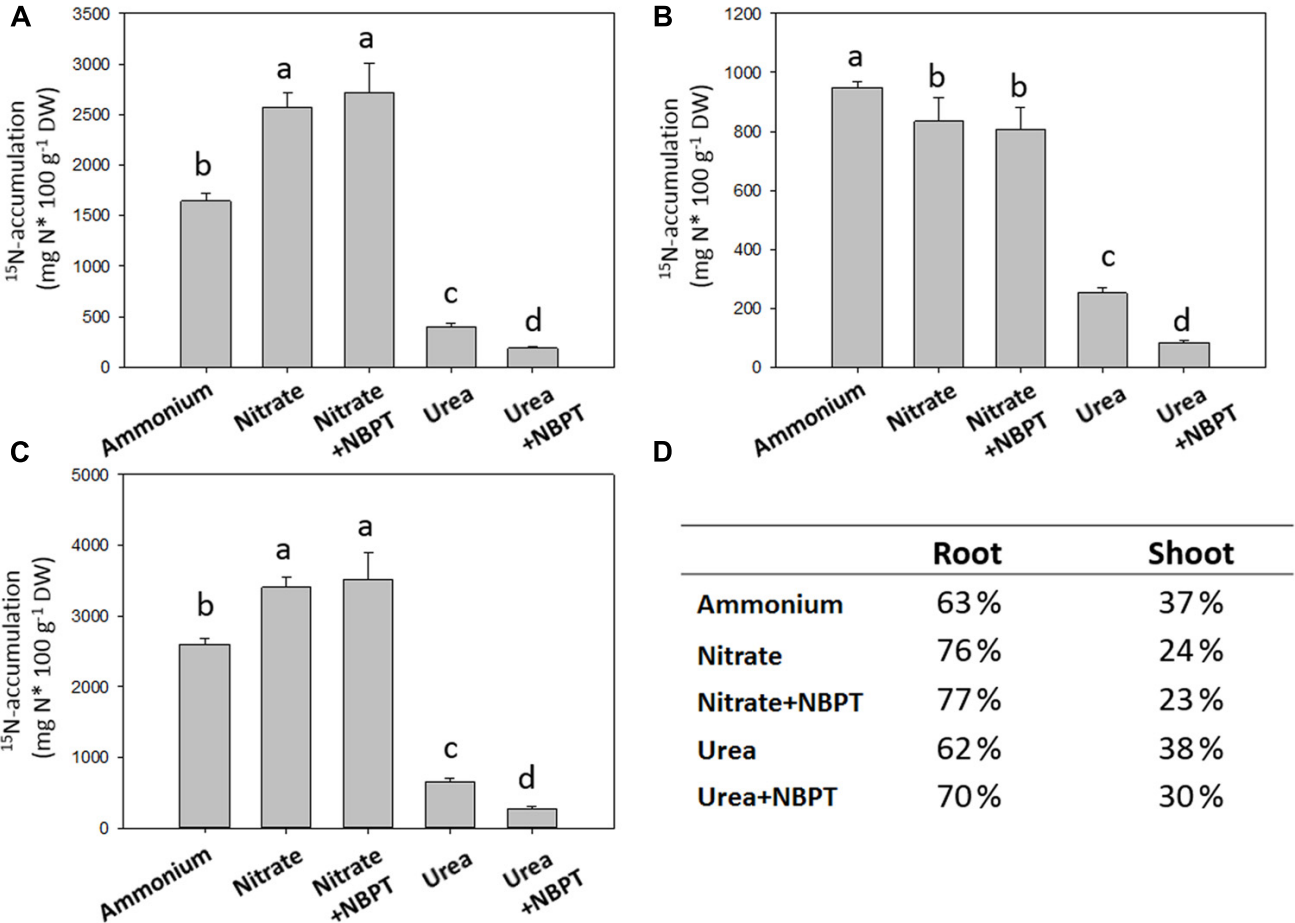
**^15^N-accumulation in maize plants.** Five-day-old maize plants were grown for 24 h in a complete nutrient solution containing N in form of ^15^N-labeled sources: 0.5 mM ^15^N-(NH_4_)_2_SO_4_ (*Ammonium* treatment); 0.5 mM ^15^N-Ca(NO_3_)_2_ (*Nitrate* treatment); or 0.5 mM ^15^N-urea (*Urea* treatment). ^15^N-Nitrate or ^15^N-urea were also provided in presence of 0.897 μM urease inhibitor NBPT (*Nitrate* + *NBPT* and *Urea* + *NBPT* treatments, respectively). The amount of ^15^N-accumulated in roots **(A)**, in shoots **(B)**, and in the whole plant **(C)** is shown. **(D)** The percentages of ^15^N-accumulation in shoot and root tissues are reported. Data are means + SD of three independent experiments and different letters above the bars indicate statistically significant differences (Student–Newman–Keuls method ANOVA, *n* = 3, *P* < 0.05).

In comparison to *Urea* treated plants, maize plants grown under nitrate or ammonium showed higher ^15^N accumulation (about 3500 mg ^15^N 100 g^-1^ DW and 2600 mg ^15^N 100 g^-1^ DW were accumulated under *Nitrate* and *Ammonium* treatments, respectively; **Figure 3C**). However, a higher percentage of ^15^N was translocated to the shoots of *Ammonium* and *Urea* treated plants with respect to *Nitrate* fed plants (**Figures 3B,D**).

In *Urea* treated plants, ^15^N accumulation was strongly reduced by about 60% in the presence of NBPT (*Urea* + *NBPT* treatment), with a reduction of 52% in roots and 67% in shoots (**Figures 3A,B**). Moreover, the presence of urease inhibitor in urea treated plants even impaired the root-to-shoot translocation of N (**Figure 3D**).

On the other hand, in plants fed with nitrate no effect of the urease inhibitor NBPT was observed on ^15^N accumulation and root-to-shoot translocation (**Figure 3**).

### Effects of NBPT on Urea Uptake Rate and Internal Concentrations of Urea and Ammonium in Maize Plants

**FIGURE 4:**
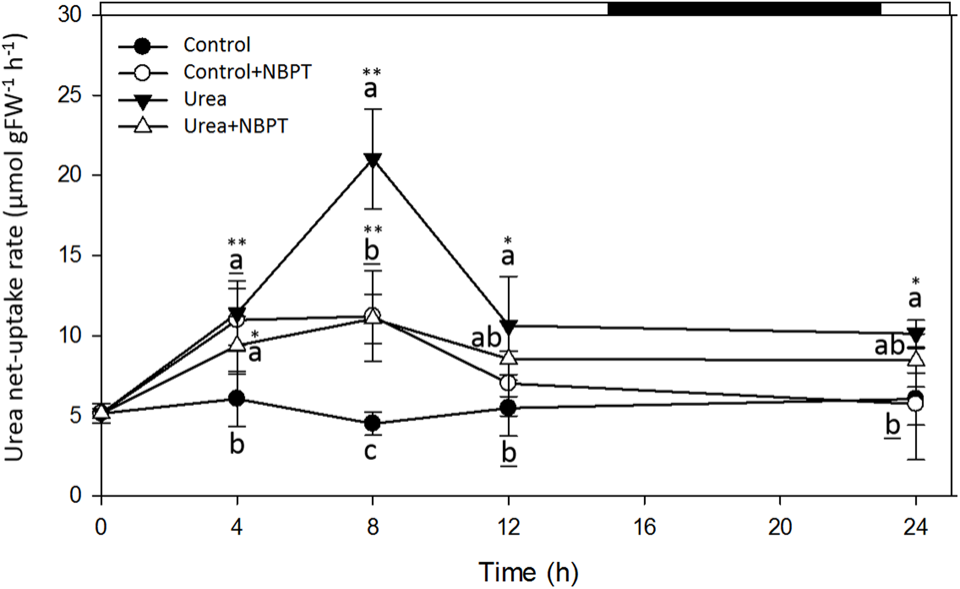
**Effect of NBPT on the development of the high-affinity urea uptake in maize roots.** Five-day-old maize plants were exposed up to 24 h to a nutrient solution supplied with 0.5 mM urea in presence or absence of 0.897 μM NBPT (*Urea* + *NBPT* treatment or *Urea* treatment, respectively) or to a N-free nutrient solution, with or without NBPT (*Control* + *NBPT* treatment or *Control* treatment, respectively). Urea net uptake rate was measured by depletion from a solution containing 0.2 mM urea in a 10-min assay using plants collected at the indicated times during the treatment. *Filled circles, Control* treatment; *open circles, Control* + *NBPT* treatment; *filled triangles, Urea* treatment; *open triangles, Urea* + *NBPT* treatment; *white and black bars*, light and dark period, respectively. Data are means ± SD; *letters* refer to statistically significant differences within each time point among three independent biological replicates, *underlined letters* refer to overlapping data not significantly different among treatments; *asterisks* refer to statistically significant differences between each time point and time zero [Student–Newman–Keuls method ANOVA, *n* = 3, ^∗^*P* < 0.05, ^∗∗^*P* < 0.001].

To investigate the effect of the urease inhibitor NBPT on the urea net uptake rate in maize, a time course experiment was performed (**Figure 4**).

During the 24 h of treatment, no significant modulation of urea uptake was observed for roots of *Control* plants (no N-source added), while *Urea* treated plants showed a transient induction of uptake rate, with a peak after 8 h of treatment.

A different behavior was observed when plants were treated with NBPT. In control plants, a weak but significant induction of urea uptake was observed after 4–8 h of treatment in presence of the inhibitor (*Control* + *NBPT*). A similar behavior was observed for plants treated with urea and NBPT; however, the presence of the inhibitor in the nutrient solution severely limited the development of a higher uptake capacity.

**FIGURE 5:**
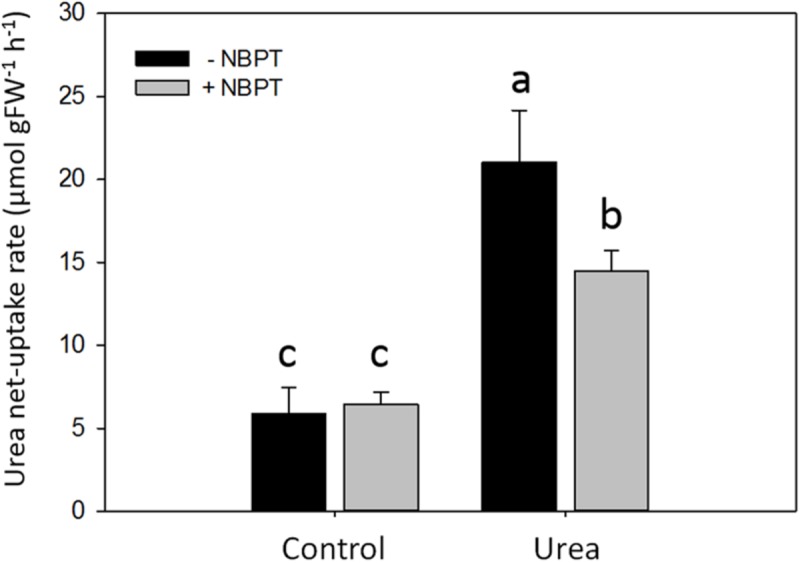
**Direct effect of NBPT on the high affinity urea uptake of maize roots.** Five-day-old maize plants were exposed for 8 h to a nutrient solution supplied with 0.5 mM urea (*Urea* treatment) or to a N-free nutrient solution (*Control* treatment). Urea net uptake was measured by depletion from a solution containing 0.2 mM urea with or without 0.897 μM NBPT. Data are means + SD of three independent biological replicates, letters refer to statistically significant differences (Student–Newman–Keuls method ANOVA, *n* = 3, *P* < 0.05).

In order to evaluate a possible direct effect of NBPT on the urea transport system, net uptake rate was measured in plants pre-treated for 8 h without (*Control*) or with urea (*Urea* treatment) and the urease inhibitor was added only to the assay solution for 10 min (**Figure 5**). In *Control* plants urea uptake was not impaired by NBPT. On the other hand, the inhibitor significantly reduced the uptake capacity of *Urea*-treated plants.

**FIGURE 6:**
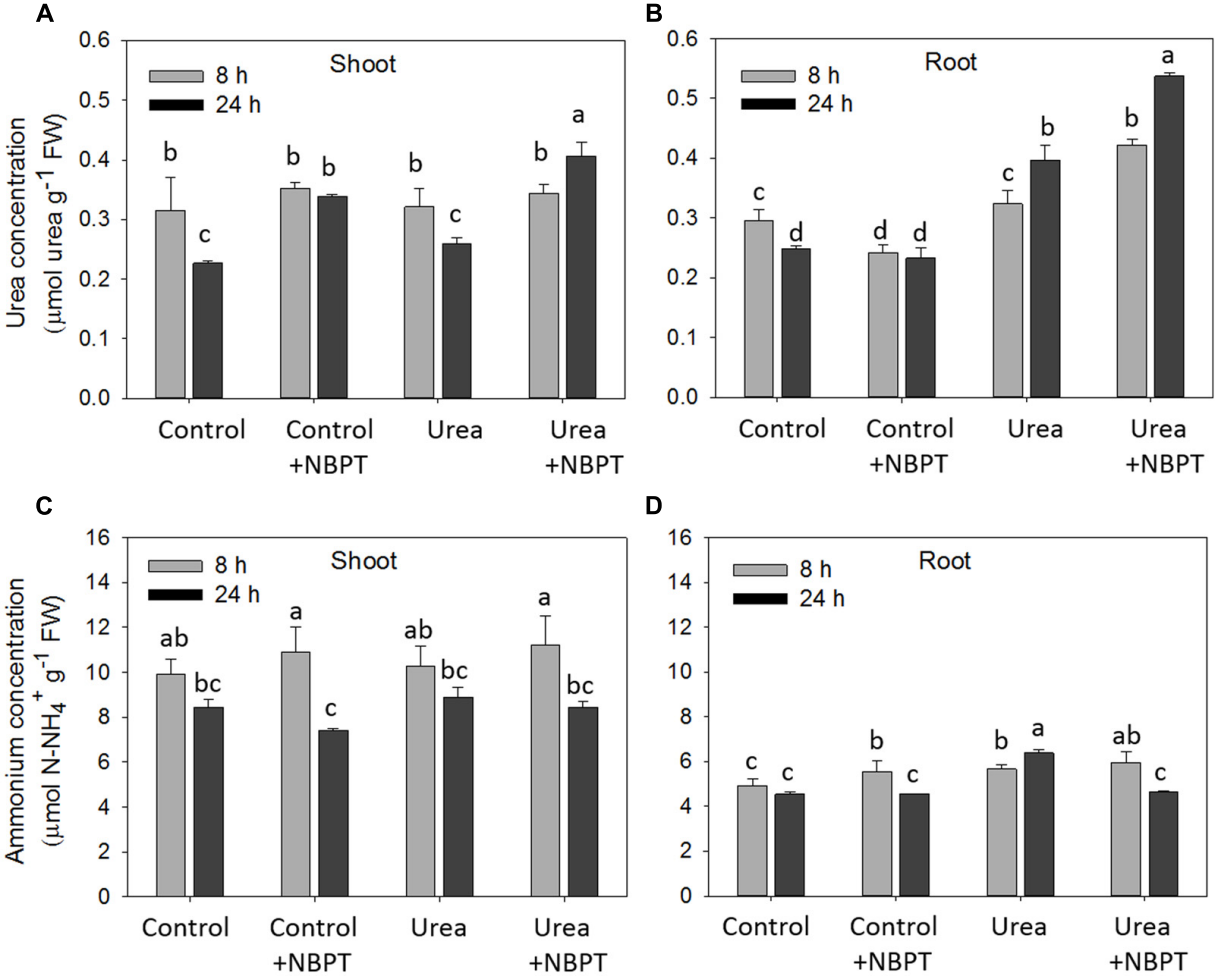
**Effect of NBPT on the urea and ammonium concentrations in shoots and roots of maize.** Five-day-old maize plants were exposed up to 24 h to a nutrient solution supplied with 0.5 mM urea in presence or absence of 0.897 μM NBPT (*Urea* + *NBPT* treatment or *Urea* treatment, respectively) or exposed to a nutrient solution without addition of any N source, with or without NBPT (*Control* + *NBPT* treatment or *Control* treatment, respectively). **(A)** Urea concentration in shoots; **(B)** urea concentration in roots; **(C)** ammonium concentration in shoots; **(D)** ammonium concentration in roots (Student–Newman–Keuls method ANOVA, *n* = 3, *P* < 0.05).

The concentration of urea and ammonium in shoots and roots was measured after 8 and 24 h of urea treatment (**Figure 6**).

As compared to *Control* plants (no N supply), plants exposed to urea showed a higher urea concentration in roots after 24 h of treatment while ammonium concentration increased already after 8 h (**Figures 6B,D**). *Control* and *Urea*-treated plants showed comparable concentrations of urea and ammonium in shoots (**Figures 6A,C**).

The addition of NBPT to the urea-containing nutrient solution caused a significant increase in urea concentration both in roots and shoots (**Figures 6A,B**). On the other hand, ammonium concentration decreased in roots due to the presence of NBPT while it remained unchanged in leaves (**Figure 6D**).

### Transcriptional Response of Genes Involved in Urea Acquisition to Urea and NBPT

**FIGURE 7:**
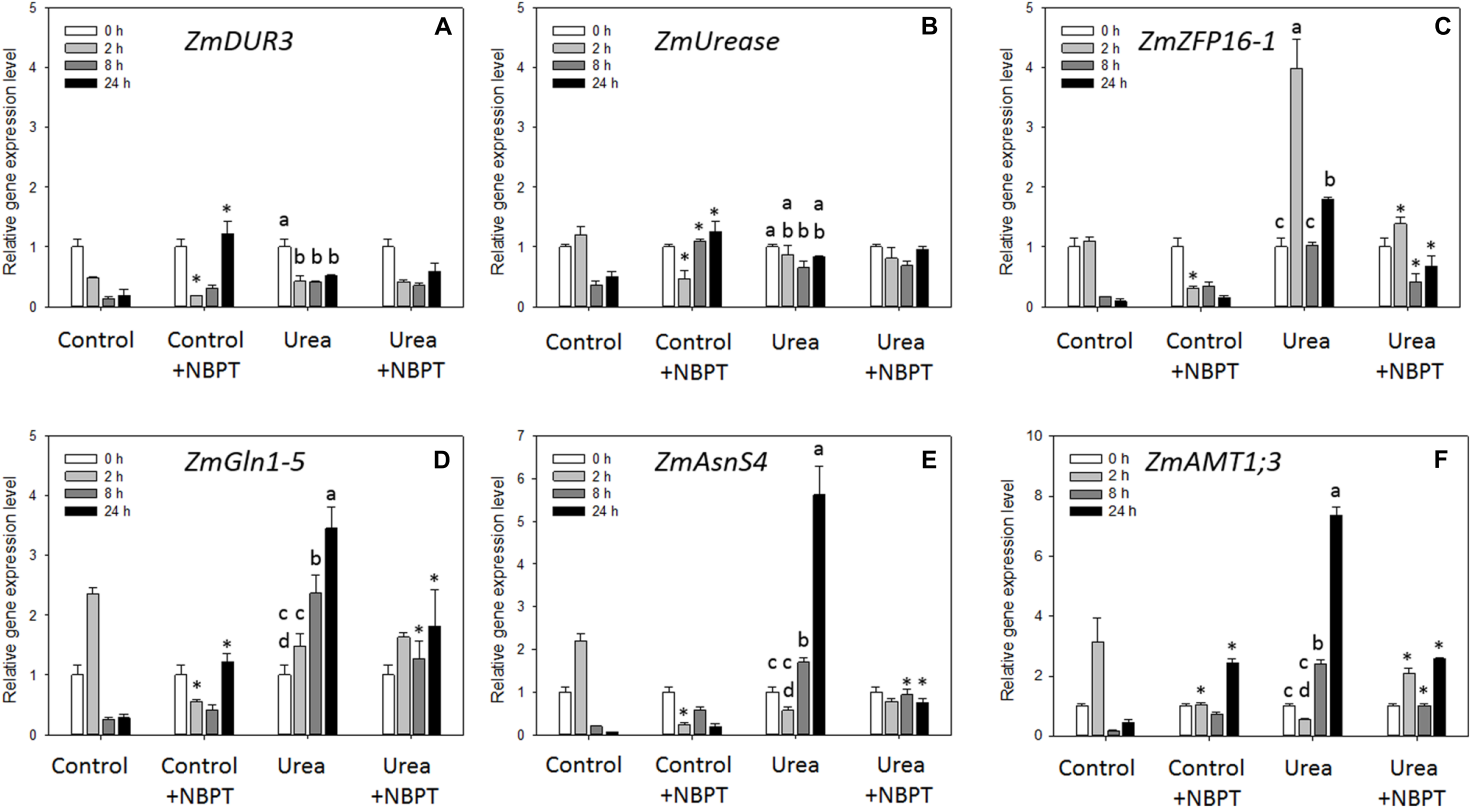
**Real-time RT-PCR analyses of gene transcript levels in maize roots.** Growth conditions as in **Figure 4**. Root samples were harvested at the beginning of the treatment (*t* = 0 h) and after 2, 8, and 24 h. Analyzed genes encode: high-affinity urea transporter (*ZmDUR3*, **A**); urease enzyme (*ZmUrease*, **B**); zinc finger protein transcription factor (*ZmZFP16-1*, **C**); cytosolic glutamine synthetase (*ZmGln1-5*, **D**); AsnS4 (*ZmAsnS4*, **E**); AMT (*ZmAMT1;3*, **F**). Gene mRNA levels were normalized with respect to the mean transcript level of the housekeeping gene *ZmGAPDH*; relative changes in gene transcript levels were calculated on the basis of the mean transcript level of *ZmGAPDH* in roots of *Control* plants at 0 h (relative gene expression = 1). Data are means of three independent biological replicates +SD; *letters* refer to statistically significant differences within the *Urea*-treatment; *asterisks* refer to statistically significant differences between each time point of treatment containing NBPT and those without (*Control* + *NBPT* treatment was compared with *Control* treatment, while *Urea* + *NBPT* treatment was compared with *Urea* treatment) at the same time (Student–Newman–Keuls method ANOVA, *n* = 3, ^∗^*P* < 0.05).

With the aim to verify if the physiological effect of NBPT on urea uptake might be related to changes at transcriptional level, the expression profile of genes involved in urea acquisition was monitored during the 24 h of treatment by real time RT-PCR (**Figure 7**).

The presence of urea in the nutrient solution (*Urea* treatment) led to a down-regulation of the *ZmDUR3* gene expression and, although to a lesser extent, also of *ZmUrease* gene (**Figures 7A,B**). A completely different behavior was observed for *ZmGln1-5, ZmAsnS4, ZmAMT1;3* and *ZmZFP16-1*, which were up-regulated by urea (maximum fold changes from 3 to 7, **Figures 7C–F**). Concerning the first three genes, a gradual increase in expression was observed during the 24 h of urea treatment (**Figures 7D–F**), while a rapid modulation of the *ZmZFP16-1* transcription factor occurred, showing a significant overexpression already after 2 h of treatment followed by a down-regulation at 8 h (**Figure 7C**).

In presence of urea and NBPT (*Urea* + *NBPT*), the expression of *ZmDUR3* and *ZmUrease* genes did not change with respect to what observed for *Urea* treatment (**Figures 7A,B**). On the other hand, NBPT severely limited the expression of the other genes tested: *ZmGln1-5, ZmAsnS4, ZmAMT1;3*, and *ZmZFP16-1*. In comparison to *Urea* treated roots, *Urea* + *NBPT* treated roots showed a down-regulation of *ZmZFP16-1* gene expression already after 2 h, while the expression of *ZmGln1-5, ZmAsnS4*, and *ZmAMT1;3* was down-regulated only after 8 h of exposure to the inhibitor (**Figures 7C,F**). Expression of *ZmAMT1;1*, that has been proposed to code for a protein with function similar to that of ZmAMT1;3 (Gu et al., 2013), was not modulated by *Urea* or *Urea* + *NBPT* treatments (Supplementary Table S3).

Also plants not exposed to any source of N showed slightly altered expression of all six genes when exposed to NBPT (*Control* + *NBPT* plants in comparison to *Control* plants), with a general down-regulation after 2 h of treatment (**Figures 7A–F**).

### Effects of NBPT on *Arabidopsis* Growth

**FIGURE 8:**
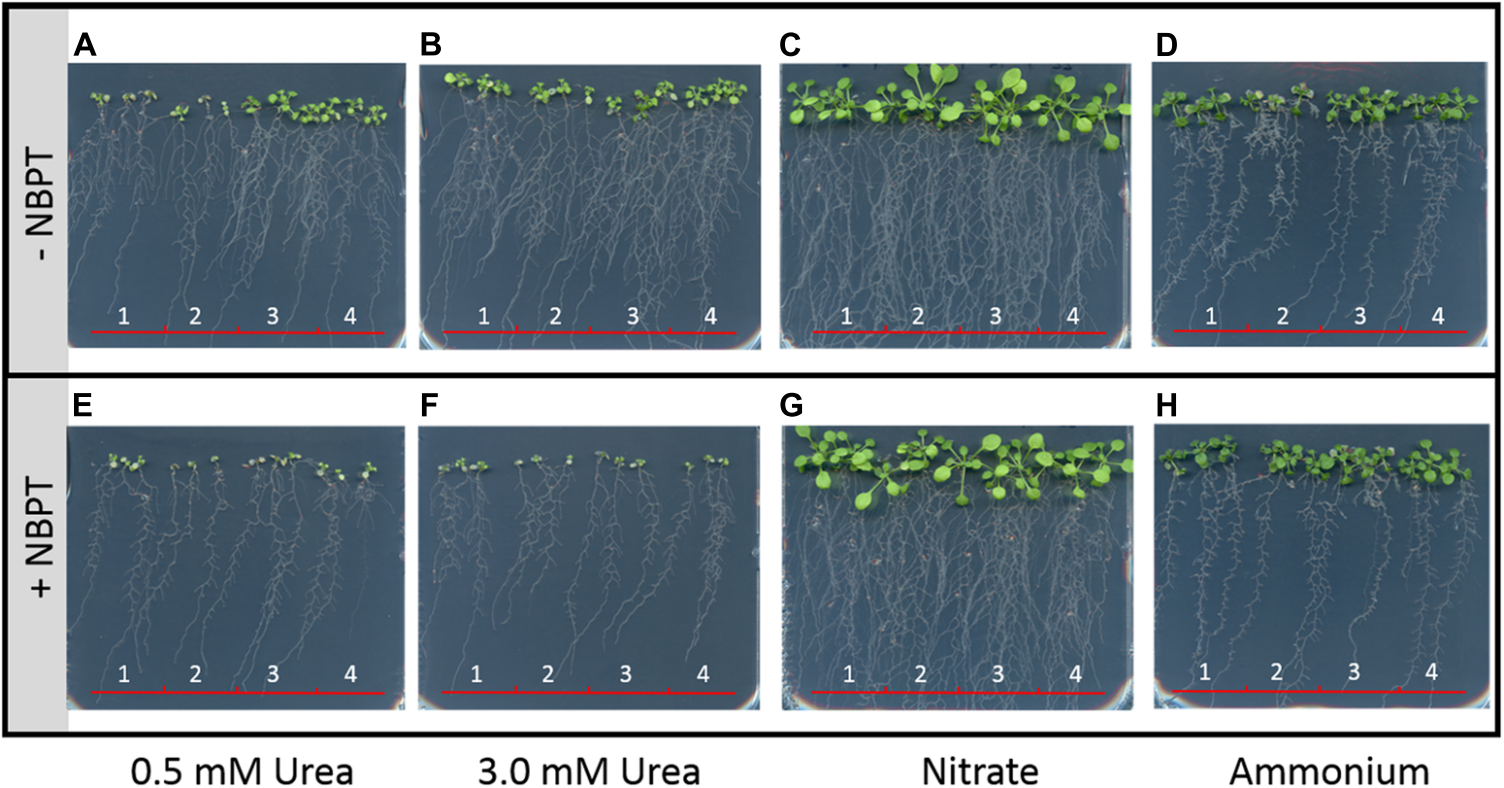
**Effect of NBPT on *Arabidopsis* growth.** Plants were grown for 18 days on sterile half strength MS agar medium supplied with 1 μM NiCl_2_ and 50 μM NO_3_^-^. For the treatments nitrogen was supplied in form of 0.5 mM urea (plates **A** and **E**), 3.0 mM urea (plates **B** and **F**), 0.5 mM Ca(NO_3_)_2_ (plates **C** and **G**), or 0.5 mM (NH_4_)_2_SO_4_ (plates **D** and **H**); 0.897 μM urease inhibitor NBPT was added where indicated (+NBPT). Numbers indicate the *Arabidopsis* lines: *1*, wild type (Col-0); *2, dur3-*knockout mutant line (*atdur3-3*); *3*, wild type overexpressing *ZmDUR3* (Col-0 + *ZmDUR3*); *4*; *atdur3-3* line overexpressing *ZmDUR3* (*atdur3-3* + *ZmDUR3*). Three independent experiments were carried out, representative pictures of one experiment are reported. FWs of *Arabidopsis* shoots are provided in Supplementary Table S4.

In order to further test the effect of NBPT on urea transport system, *ZmDUR3* transformed *Arabidopsis* plants were used. The *atdur3-3* mutant is defective in the endogenous urea transporter AtDUR3, showing a slow growth and chlorotic leaves when supplied with 0.5 mM urea as the sole N source (Kojima et al., 2007; Zanin et al., 2014; **Figure 8A**); in these conditions wild type plants grew slightly better than *atdur3-3* plants (**Figure 8A**). On the other hand, the two *ZmDUR3*-overexpressing lines showed better shoot development and root proliferation as compared to wild type plants (**Figure 8A**). At high urea concentration (3 mM urea), all four *Arabidopsis* lines were able to grow well and to develop a suitable root system, without showing any appreciable difference among them (**Figure 8B**). When NBPT was added to urea-containing agar medium (0.5 or 3 mM urea), growth of all four lines was drastically compromised (**Figures 8E,F**; Supplementary Table S4).

Interestingly, when *Arabidopsis* lines were grown on agar plate containing ammonium or nitrate as a sole N source, no significant difference among the four lines was observed either in absence or presence of NBPT (**Figures 8C,D,G,H**; Supplementary Table S4).

## Discussion

In soil urea is rapidly hydrolyzed to ammonium and carbon dioxide by the action of microbial ureases. It has been calculated that a significant portion of applied urea (more than 50%) is lost through ammonia volatilization (Terman, 1979), decreasing the amount of N available for plant nutrition. A common agronomic practice to increase the efficiency of urea-based fertilizers consists into applying urease inhibitors, such as NBPT in conjunction with urea fertilizers (Watson, 2005).

In this work, the effect of NBPT on the root capacity to acquire urea was studied. In agreement with previous observations (Zanin et al., 2015a), data on biomass production and root proliferation indicated that maize plants are able to grow in presence of urea as a sole N source. However, the presence of NBPT in the urea-containing nutrient solution negatively affected plant growth (**Figures 1** and **2**; Supplementary Table S1). In several plants, like sorghum, wheat, ryegrass, or pea, visible symptoms of NBPT toxicity (e.g., leaf necrosis) have been reported (Krogmeier et al., 1989; Watson and Miller, 1996; Artola et al., 2011; Cruchaga et al., 2011). This effect has been ascribed to an elevated urea concentration within the plant tissue due to a reduced endogenous urease activity. In maize seedlings, no yellowing or necrosis were observed, similarly to spinach plants (Cruchaga et al., 2011). This result might be due to the short time of exposure (7 days) or, as suggested for spinach, to the high N assimilation capacity in the leaves of this plant species.

To evaluate if the observed growth reduction caused by NBPT might be related to a decreased urea influx we fed plants with ^15^N-labeled sources for 24 h (**Figure 3**). Data of ^15^N-accumulation (**Figure 3**), as well as urea and ammonium concentrations in maize tissues (**Figures 6B,D**), confirmed that maize plants are able to use urea as a source of N, although less efficiently when compared with the inorganic sources, ammonium or nitrate (Mérigout et al., 2008a,b; **Figure 3**). In accordance with data from pea (Cruchaga et al., 2011), our results showed that in shoots and roots the accumulation of ^15^N derived from urea was strongly reduced by the presence of NBPT (**Figure 3**), while having no effect on accumulation of nitrate-derived ^15^N. The inhibitor NBPT also affected the capability of maize plants to use the ureic N as demonstrated by a reduction in ammonium concentration in maize roots (**Figure 6D**). These results suggest that NBPT action was directed toward urea acquisition mechanisms rather than being due to a general effect on N nutrition. This idea was reinforced by growing *Arabidopsis* plants on agar plates (see below) since plant growth was not limited by NBPT under ammonium or nitrate nutrition (**Figure 8**; Supplementary Table S4).

In order to better characterize the effect of NBPT on urea transport in maize, the net uptake rate of urea was analyzed under low urea external concentration, mimicking the condition conceivably present in the soil solution (**Figure 4**). As previously described (Zanin et al., 2015a), a transient induction of the urea uptake rate was observed in *Urea* treated plants; however, this increase was severely limited in presence of NBPT (**Figure 4**). A significant reduction of the urea uptake rate was also observed when *Urea* treated plants were briefly exposed (10 min) to the inhibitor in the assay solution (**Figure 5**).

Taken together these results indicate that NBPT negatively affects the capacity of maize plants to acquire urea, at least partially through a direct action on the high affinity uptake system.

Inhibition of uptake could be due to a competition between NBPT and urea; the urea analog thiourea was shown to inhibit urea uptake at equimolar concentration in *Xenopus laevis* oocytes expressing *OsDUR3* (Wang et al., 2012). However, it should be noted that the concentration of NBPT used in the present work was 500 times lower than that of urea. Alternatively binding to DUR3 might occur slowing its activity. Further studies are needed to shed light on these aspects.

The recent characterization of DUR3 as an urea transporter among cultivated plants (Wang et al., 2012; Zanin et al., 2014) and the identification of the *Arabidopsis* DUR3 as the major component of the high affinity uptake system from the soil solution (Kojima et al., 2007) suggest a key role of this transporter on the acquisition of external urea when supplied at low concentration.

*ZmDUR3* expression was not induced in roots when urea was present in the external solution (**Figure 7A**), confirming previous observations (Zanin et al., 2014). Also, the treatment with NBPT did not alter its expression during the time span of 24 h. Some authors have reported as the availability of N could repress the expression of *DUR3* genes (Kojima et al., 2007; Arkoun et al., 2012) while prolonged N starvation positively regulated its expression (Zanin et al., 2014, 2015a; Bohner et al., 2015; Liu et al., 2015). In order to provide a more detailed assessment of the interaction of NBPT with the high affinity urea transport system, we performed a growth test on agar medium using lines of *Arabidopsis* overexpressing *ZmDUR3* and *dur3*-knockout (**Figure 8**). Interestingly, on agar medium containing urea plus NBPT, the growth of all the *Arabidopsis* lines tested (Col-0, *atdur3*-*3* mutant line and the *ZmDUR3*-overexpressing lines) was compromised (**Figures 8E,F**; Supplementary Table S4). Even those lines overexpressing *ZmDUR3* were unable to survive in presence of the urease inhibitor, irrespective of the urea concentration of the nutrient solution (either 0.5 or 3 mM urea; **Figures 8E,F**; Supplementary Table S4). These data suggest that not only the high affinity transport system, but the N-urea acquisition machinery could be affected by NBPT.

In some plants, like pea and wheat, the treatment with NBPT led to a reduced urease activity, lower ammonium content and to an altered amino acid profile; on the other hand an over-accumulation of urea in plant tissue was measured (Artola et al., 2011; Cruchaga et al., 2011).

These observations were confirmed in the present work; in fact, roots and shoots of maize plants treated with urea and NBPT showed an accumulation of urea with a concomitant reduction of ammonium concentration in the roots. For this reason, we evaluated the expression profile of metabolic enzymes, which are involved in the primary steps of urea assimilation in the roots.

Although some authors reported a lowered activity of plant urease as a consequence of NBPT treatment (Cruchaga et al., 2011), no significant change in the expression of a gene encoding for urease was recorded in maize roots (**Figure 7B**). Under urea nutrition, the hydrolysis of urea by urease releases high amounts of ammonium, which is assimilated into amino acids, such as glutamine and asparagine. In *Arabidopsis* and pea urea nutrition led to an accumulation of high levels of these two amino acids in the roots (Mérigout et al., 2008a; Cruchaga et al., 2011), while their contents and the activity of glutamine-synthetase were significantly reduced upon NBPT treatment (Artola et al., 2011; Cruchaga et al., 2011). In agreement with previous observation (Zanin et al., 2015a), the exposure of maize roots to urea caused a strong induction in the expression of *ZmGln1-5* and *ZmAsnS4* genes, coding for a cytosolic glutamine-synthetase and an asparagine-synthetase, respectively. However, this induction was prevented by adding NBPT to the urea-containing nutrient solution (**Figures 7D,E**). These transcriptional data might provide a reasonable explanation for changes in glutamine and asparagine contents as well in glutamine-synthetase activity (Artola et al., 2011; Cruchaga et al., 2011).

In recent years, *ZmAMT* genes have been characterized as coding for high affinity AMTs, which differ in their spatial localization and biochemical properties (Gu et al., 2013). A gene coding for the AMT1;3 protein was shown to be responsive to ammonium with its transcripts localized in the epidermal cells of the apical root zone and in the pericycle cell layer of the stele (Gu et al., 2013). In the present study, we could show that urea nutrition induced *ZmAMT1;3* expression (**Figure 7F**) following a pattern similar to those observed for *ZmGln1-5* and *ZmAsnS4* (**Figures 7D,E**). In accordance with the evidence reported by Gu et al. (2013), this result might indicate the involvement of ZmAMT1;3 in the redistribution of N derived from urea hydrolysis as well as in the re-acquisition of cytosolic ammonium lost by diffusion through plasma membrane of epidermal cells. As previously observed for the two enzymes, also the expression of *ZmAMT1;3* was reduced by the presence of NBPT in the urea-containing nutrient solution, possibly as a consequence of a lower ammonium production (**Figure 6**) by the inhibited urease (Artola et al., 2011; Cruchaga et al., 2011).

Recently, microarray analyses in maize roots revealed a transcription factor responsive to urea nutrition, a zinc finger protein ZFP16-1 (Zanin et al., 2015a). Its homolog in *Arabidopsis* (ZAT12, gene ID: AT5G59820) was likewise responsive to urea (Mérigout et al., 2008a) and was found to be upregulated under stress conditions (e.g., H_2_O_2,_ cold, salinity) playing a key role in tolerance to these stresses (Rizhsky et al., 2004; Davletova et al., 2005). Furthermore, ZAT12 has been reported to be specifically induced by spermine (Mitsuya et al., 2009); this polyamine is implicated in a wide range of plant growth and developmental processes (Pang et al., 2007). The oxidation of spermine might in turn release H_2_O_2_ which acts as signal molecule to induce genes involved in the H_2_O_2_ signaling pathway (Mitsuya et al., 2009), like cellular defense responses to biotic and abiotic stresses (Walters, 2003). In the present work we could show that *ZmZFP16-1* was upregulated already after 2 h of treatment with urea; however, this induction was strongly limited by the presence of NBPT in the nutrient solution (**Figure 7C**). This result suggests an involvement of this transcription factor in the overall mechanism of ureic N assimilation and redistribution in plants. As an early responsive element, this transcription factor might play a key role to activate the pathway for urea assimilation and in turn activate the inducible acquisition of urea in plants.

## Conclusion

The results of the present work show that the presence of NBPT in the root external solution can lead to an inhibition of urea uptake mechanisms and prevent induction of genes involved in its assimilation, besides the well-documented effect on urease activity.

Other urease inhibitors, having structural analogy to urea, might affect urea acquisition in a way similar to NBPT. Although different experimental approaches (e.g., time of exposure to urea and/or to the inhibitor) and plant species used may render difficult a generalization, it has been shown (Arkoun et al., 2013) that phosphorodiamidate (PPD) limited ^15^N accumulation, glutamine-synthetase activity and decreased shoot and root amino acid content in rapeseed.

The present study provides a basis for better understanding of the overall influence of urease inhibitors, like NBPT, whose effects might limit the efficiency of urea-containing fertilizers. This would help developing new strategies and/or products able to better reconcile the need to preserve urea availability in the soil and the functionality of urea acquisition system in crops.

## Author Contributions

LZ and NT acquired and analyzed the data. AZ and ZV carried out the ^15^N- analyses. LZ, NT, RP designed and oversaw the research; LZ, NT, AZ, ZV, and RP wrote the article. All authors read and approved the final manuscript.

## Conflict of Interest Statement

The authors declare that the research was conducted in the absence of any commercial or financial relationships that could be construed as a potential conflict of interest.

## Footnotes

1 http://www.r-project.org/

2 http://www.dr-spiess.de/qpcR.html

## Abbreviations

AMT: ammonium transporter
AsnS4: asparagine synthetase 4
DUR3: high affinity urea transporter 3
DW: dry weight
FW: fresh weight
Gln1-5: glutamine synthetase 1-5
N: nitrogen
NBPT: *N*-(*n*-butyl) thiophosphoric triamide
ZFP16-1: zinc finger protein
