# Supplementary material for: The Urease Inhibitor NBPT Negatively Affects DUR3-mediated Uptake and Assimilation of Urea in Maize Roots
Source: Front Plant Sci. 2015 Nov 19;6:1007. doi: 10.3389/fpls.2015.01007 (PMC4652015; doi:10.3389/fpls.2015.01007)
Supplement: Supplementary file 1 [file Table_1.DOCX]

Supplementary Material

**The urease inhibitor NBPT negatively affects DUR3-mediated uptake and assimilation of urea in maize roots**

**Laura Zanin*, Nicola Tomasi, Anita Zamboni, Zeno Varanini, Roberto Pinton**

***Correspondence:** Laura Zanin, [laura.zanin@uniud.it](mailto:laura.zanin@uniud.it)

# Supplementary Table

**Supplementary Table 1. Morphometric evaluation of maize roots in response to nitrogen treatments.** Five-days old maize plants were grown hydroponically for 7 days in nutrient solution containing nitrogen in form of: 0.5 mM ammonium sulphate (*Ammonium* treatment); 0.5 mM calcium nitrate (*Nitrate* treatment); 0.5 mM urea in presence or absence of NBPT (*Urea+NBPT* treatment or *Urea* treatment, respectively); 0.5 mM urea and 0.5 mM ammonium sulphate (*Urea+Ammonium* treatment); 0.5 mM urea and 0.5 mM calcium nitrate (*Urea+Nitrate* treatment). Control plants were grown in nitrogen starvation with or without NBPT (*Control+NBPT* treatment or *Control* treatment, respectively). The analyses of root systems were performed using “GiA Roots” software (Galkovskyi *et al.*, 2012). Trait descriptions are following (Galkovskyi *et al.*, 2012): *Maximum Number of Roots*, after sorting the number of roots crossing a horizontal line from smallest to largest, the maximum number is considered to be the 84^th^-percentile value (one standard deviation); *Median Number of Roots*, the result of a vertical line sweep in which the number of roots that crossed a horizontal line was estimated, and then the median of all values for the extent of the network was calculated; *Network Area* (cm^2^), the number of network pixels in the image; *Network Perimeter* (cm), the total number of network pixels connected to a background pixel; *Network Surface Area* (cm^2^), the sum of the local surface area at each pixel of the network skeleton, as approximated by a tubular shape whose radius is estimated from the image; *Network Length* (cm), the total number of pixels in the network skeleton; the “GiA Roots” software converted *pixel* data in *cm* unit. Data are means ± SD based on three independent biological replicates (*small letters* refer to statistically differences among the mean, Student–Newman–Keuls method ANOVA, n=3, P<0.05).

|  | **Maximum Number of Roots** | | | **Median Number of Roots** | | | **Network Area** | | | **Network Perimeter** | | | **Network Surface Area** | | | **Network Length** | | |
| --- | --- | --- | --- | --- | --- | --- | --- | --- | --- | --- | --- | --- | --- | --- | --- | --- | --- | --- |
|  |  |  |  |  |  |  |  |  |  |  |  |  |  |  |  |  |  |  |
| *Control* | 33.5 | ± | 5.4 b | 16.0 | ± | 4.6 a | 27.2 | ± | 5.7 c | 1869.4 | ± | 450.5 c | 96.5 | ± | 21.3 c | 970.3 | ± | 246.7 c |
| *Control+NBPT* | 32.3 | ± | 2.6 b | 17.3 | ± | 1.9 a | 28.8 | ± | 3.3 c | 2018.2 | ± | 209.5 c | 102.8 | ± | 12.2 c | 983.1 | ± | 119.3 c |
| *Ammonium* | 19.0 | ± | 2.1 c | 5.1 | ± | 0.7 b | 13.1 | ± | 1.4 d | 870.4 | ± | 70.0 d | 47.2 | ± | 4.9 d | 426.2 | ± | 33.7 d |
| *Nitrate* | 44.4 | ± | 3.4 a | 18.0 | ± | 0.7 a | 31.4 | ± | 3.8 c | 2320.8 | ± | 333.2 c | 113.3 | ± | 13.2 c | 1153.1 | ± | 157.8 bc |
| *Urea* | 50.3 | ± | 7.1 a | 17.5 | ± | 2.6 a | 39.8 | ± | 3.2 b | 2748.8 | ± | 85.5 b | 140.8 | ± | 11.1 b | 1309.8 | ± | 31.2 b |
| *Urea+NBPT* | 42.5 | ± | 7.2 a | 15.3 | ± | 3.6 a | 34.0 | ± | 4.7 c | 2305.5 | ± | 299.9 c | 121.2 | ± | 17.7 c | 1111.6 | ± | 162.0 bc |
| *Urea+Ammonium* | 22.0 | ± | 0.7 c | 6.2 | ± | 0.7 b | 15.6 | ± | 1.4 d | 1054.0 | ± | 79.0 d | 55.8 | ± | 4.9 d | 505.6 | ± | 39.2 d |
| *Urea+Nitrate* | 49.8 | ± | 2.8 a | 20.1 | ± | 2.2 a | 50.5 | ± | 3.0 a | 3264.3 | ± | 110.1 a | 181.1 | ± | 10.8 a | 1580.7 | ± | 47.3 a |
