# Supplementary material for: The Urease Inhibitor NBPT Negatively Affects DUR3-mediated Uptake and Assimilation of Urea in Maize Roots
Source: Front Plant Sci. 2015 Nov 19;6:1007. doi: 10.3389/fpls.2015.01007 (PMC4652015; doi:10.3389/fpls.2015.01007)
Supplement: Supplementary file 2 [file Table_2.DOCX]

Supplementary Material

**The urease inhibitor NBPT negatively affects DUR3-mediated uptake and assimilation of urea in maize roots**

**Laura Zanin*, Nicola Tomasi, Anita Zamboni, Zeno Varanini, Roberto Pinton**

***Correspondence:** Laura Zanin, [laura.zanin@uniud.it](mailto:laura.zanin@uniud.it)

# Supplementary Tables

**Supplementary Table 2. List of primers used to perform real time RT-PCR analyses, as described in Materials and Methods section.**

| **Transcript_ID** | **Descriptions** |  | **Primers (5'--3')** | **PCR Efficiency** |
| --- | --- | --- | --- | --- |
| AC202439.3_FGT006 | Urea transporter | For | CCTCAATCTGGTGGGTGTCT | 81% |
|  | (DUR3) | Rev | ATTGGCCTTTCTCCACAGC |  |
| GRMZM2G461569_T01 | Urease | For | ATGCTGATGGTTTGTCACCA | 86% |
|  | (Ure) | Rev | GCAATATGTCCTCAGCAGCA |  |
| GRMZM2G001205_T01 | C_2_H_2_-type zinc finger family protein | For | GTGGCTCGACCTCAACTACC | 82% |
|  | (ZFP16-1) | Rev | CAGTGTCGAGGAACTGATGG |  |
| GRMZM2G046601_T01 | Glutamine synthetase | For | CGATCAAGGGTGACTGGAAC | 83% |
|  | (Gln1-5) | Rev | GCCTCCTTGATCACCTCGTA |  |
| GRMZM2G078472_T02 | Asparagine synthetase | For | CTCTACGACACGCGAGACAG | 93% |
|  | (AsnS4) | Rev | CGTTCAGCGCCTTCATCTCG |  |
| GRMZM2G028736_T01 | Ammonium transporter | For | TTCCTGGCGCTCAACAAGAT | 93% |
|  | (AMT1;3) | Rev | CTCAAGCTCAAGTCGTCGTC |  |
| GRMZM2G046804_T07 | Glyceraldehyde-3-phosphate dehydrogenase | For | CCTGCTTCTCATGGATGGTT | 100% |
|  | (GAPDH) | Rev | TGGTAGCAGGAAGGGAAGCA |  |
| GRMZM2G152466_T01 | Tubulin alpha | For | AGGTCATCTCATCCCTGACG | 98% |
|  | (TUA) | Rev | TGAAGTGGATCCTCGGGTAG |  |
