# Supplementary material for: The Urease Inhibitor NBPT Negatively Affects DUR3-mediated Uptake and Assimilation of Urea in Maize Roots
Source: Front Plant Sci. 2015 Nov 19;6:1007. doi: 10.3389/fpls.2015.01007 (PMC4652015; doi:10.3389/fpls.2015.01007)
Supplement: Supplementary file 3 [file Table_3.DOCX]

Supplementary Material

**The urease inhibitor NBPT negatively affects DUR3-mediated uptake and assimilation of urea in maize roots**

**Laura Zanin*, Nicola Tomasi, Anita Zamboni, Zeno Varanini, Roberto Pinton**

***Correspondence:** Laura Zanin, [laura.zanin@uniud.it](mailto:laura.zanin@uniud.it)

# Supplementary Tables

**Supplementary Table 3. Real-time RT–PCR analyses of gene transcript levels in maize roots.** Growth conditions as in **Fig. 4**. Root samples were harvested after 8 hours of treatment. Analyzed genes encode ammonium transporters: *ZmAMT1;1a* (GRMZM2G175140_T01), *ZmAMT1;1b* (GRMZM2G118950_T01) and *ZmAMT1;3* (GRMZM2G028736_T01). Gene mRNA levels were normalized with respect to the mean transcript level of the housekeeping gene *ZmGAPDH*; relative changes in gene transcript levels were calculated on the basis of the mean transcript level of *ZmGAPDH* in roots of *Control* plants at 8 hour (relative gene expression = 1). Data are means of three independent biological replicates ± SD.

| Gene name | **Control** | | | **Urea** | | | **Urea+NBPT** | | |
| --- | --- | --- | --- | --- | --- | --- | --- | --- | --- |
|  |  |  |  |  |  |  |  |  |  |
| *ZmAMT1;1a* | 1.00 | ± | 0.21 | 1.10 | ± | 0.10 | 1.20 | ± | 0.18 |
| *ZmAMT1;1b* | 1.00 | ± | 0.29 | 1.36 | ± | 0.09 | 0.97 | ± | 0.28 |
| *ZmAMT1;3* | 1.00 | ± | 0.13 | 13.39 | ± | 0.80 | 5.60 | ± | 0.36 |
|  |  |  |  |  |  |  |  |  |  |
