# Supplementary material for: The Urease Inhibitor NBPT Negatively Affects DUR3-mediated Uptake and Assimilation of Urea in Maize Roots
Source: Front Plant Sci. 2015 Nov 19;6:1007. doi: 10.3389/fpls.2015.01007 (PMC4652015; doi:10.3389/fpls.2015.01007)

Supplementary Material

**The urease inhibitor NBPT negatively affects DUR3-mediated uptake and assimilation of urea in maize roots**

**Laura Zanin*, Nicola Tomasi, Anita Zamboni, Zeno Varanini, Roberto Pinton**

***Correspondence:** Laura Zanin, [laura.zanin@uniud.it](mailto:laura.zanin@uniud.it)

# Supplementary Tables

**Supplementary Table 4. Effect of NBPT on fresh weights of Arabidopsis shoots (mg shoot FW*plant^-1^, mean values ± SD) grown on plates A to H as shown in Figure 8.** Plants were grown for 18 days on sterile half strength MS agar medium supplied with 1 μM NiCl_2_ and 50 μM NO_3_^−^. For the treatments nitrogen was supplied in form of 0.5 mM urea (plates **A** and **E**), 3.0 mM urea (plates **B** and **F**), 0.5 mM Ca(NO_3_)_2_ (plates **C** and **G**) or 0.5 mM (NH_4_)_2_SO_4_ (plates **D** and **H**); 0.897 μM urease inhibitor NBPT was added where indicated (+ NBPT: plates **E**, **F**, **G** and **H**). Numbers indicate the Arabidopsis lines: *1*, wild type (Col-0); *2*, *dur3-*knockout mutant line (*atdur3-3*); *3*, wild type overexpressing *ZmDUR3* (Col-0 + *ZmDUR3*); *4*; *atdur3-3* line overexpressing *ZmDUR3* (*atdur3-3* + *ZmDUR3*). Below the table, **A x E**, **B x F**, **C x G**, **D x H** refer to analysis of variance showing the significant effect of inhibitor on shoot biomass (Student–Newman–Keuls method ANOVA, n=3, P<0.05).


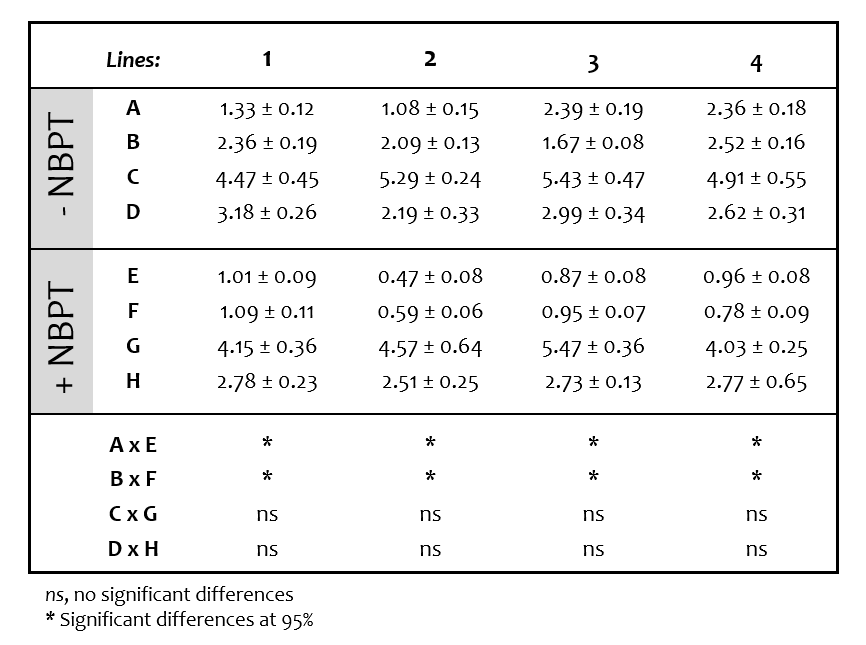

Supplement: Supplementary file 4 [file Table_4.DOCX]
